# Amphiphilic polymer conetworks based on triPEG and tetraPEG end-linked multi-armed star block copolymers

**DOI:** 10.1080/14686996.2026.2658326

**Published:** 2026-04-16

**Authors:** Constantina K. Varnava, Demetris E. Apostolides, Costas S. Patrickios, Takamasa Sakai, Florian A. Jung, Christine M. Papadakis

**Affiliations:** a Department of Chemistry, University of Cyprus, 1 University Avenue, Nicosia, Cyprus; b Department of Bioengineering, Graduate School of Engineering, The University of Tokyo, Bunkyo-ku, Japan; c TUM School of Natural Sciences, Physics Department, Soft Matter Physics Group, Technical University of Munich, Garching, Germany

**Keywords:** Hydrogels, amphiphilic polymer conetworks, reversible addition-fragmentation chain transfer (RAFT) polymerization, microphase separation

## Abstract

We present the development of amphiphilic polymer conetworks (APCNs) exhibiting long-range ordering and large domain sizes both in the bulk and in an aqueous environment, with a particular sample displaying a record domain size of 67 nm in water, almost 2 times larger than those reported for APCNs to date. These APCNs comprise equally large, end-linked multi-armed amphiphilic ‘core-first’ star block copolymers of molar masses reaching about one million g mol^−1^, prepared *via* a four-step sequential reversible addition-fragmentation chain transfer (RAFT) polymerization. The constituting star block copolymers were characterized in terms of their molar mass and composition using gel permeation chromatography and proton nuclear magnetic resonance spectroscopy, respectively, while the mesoscopic APCN structures in water and in the bulk were explored using small- and wide-angle X-ray scattering. The developed materials may find applications both in the biomedical and technology fields.

## IMPACT STATEMENT

Amphiphilic polymer conetworks based on end-linked giant amphiphilic or hydrophobic multi-armed star polymers self-organize in the bulk and in water, displaying record-long repeat distances of 50 and 67 nm, respectively.

Statement of Novelty

Amphiphilic polymer conetworks comprising giant amphiphilic star block copolymers self-assembled in water and in bulk, yielding spherical or lamellar nanophases separated at record-long repeat distances of 67 and 50 nm.

## GRAPHICAL ABSTRACT

**Figure:**
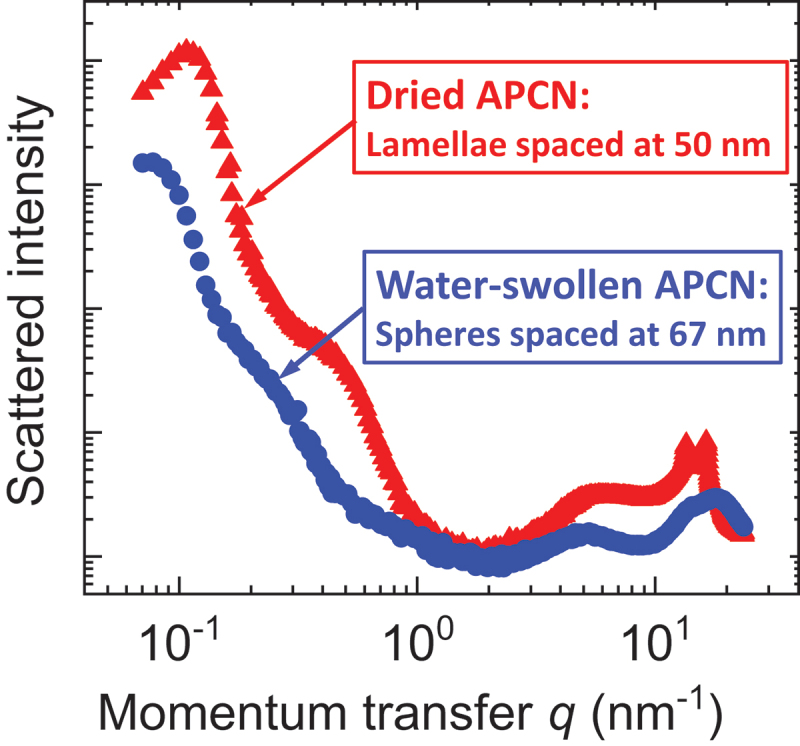

## 1. Introduction

Amphiphilic polymer conetworks (APCNs) [1–5] represent a type of crosslinked polymer network [6] consisting of hydrophilic and hydrophobic segments [7–10]. This constitution still allows them to swell in aqueous media, but less so compared to their fully hydrophilic counterparts. Other than reducing aqueous swelling, the hydrophobic segment (provided that this comprises, typically, five or more monomer repeating units) has another, more important, function: it drives the APCN system to phase separation on the nanoscale both in water and in the bulk [11]. APCNs may, consequently, be thought of as the network analogues to surfactants.

The first reports on the preparation of these materials date almost 40 years back [12–14], and APCNs are currently evaluated as matrices for a host of applications in biomedicine and technology. Efforts in the former field aim at adopting APCNs as substrates for controlled drug delivery [15] and as scaffolds for tissue engineering [16], whereas research in the latter field looks into employing APCNs as conducting electrolyte membranes for the separation of the electrodes in lithium ion batteries [17] and as ‘sponges’ for sequestering hydrophobic environmental pollutants dispersed in water [18]. However, APCNs have one major commercial application: soft contact lenses [19] consisting of hydrophobic silicone (polydimethylsiloxane, which, in addition to being hydrophobic, it is also lipophobic, soft, biocompatible and oxygen-permeable) crosslinked together with hydrophilic monomers such as 2-hydroxyethyl methacrylate or *N,N*-dimethylacrylamide [20].

APCN synthesis may readily be accomplished *via* the employment of the hydrophobic macro-crosslinker approach through which a hydrophobic polymer segment, end-capped with two vinyl groups, is free-radically copolymerized with a hydrophilic monomer [1–3,11]. This would be followed by APCN characterization and evaluation for the desired application. However, this synthetic protocol leads to materials that possess an imperfect structure in terms of the lengths of their hydrophilic segments. This segment length distribution, in turn, results in suboptimal mechanical and self-organization properties [11]. Regarding mechanical properties, upon deformation, the shorter chains would break early, depriving the material from their associated stiff interconnections. Regarding self-organization, the broadly-distributed chain sizes, in combination with the presence of the crosslinks, would not allow the system to go to minimal energy configuration, yielding blurred interfaces, and distorted/perturbed versions of the classical morphologies, *i.e*., lamellae, cylinders and spheres.

The aim here is to improve APCN structure by employing well-defined and long amphiphilic block copolymer segments, prepared using reversible deactivation radical polymerization [21]. Furthermore, several of these block copolymer segments were interconnected into a very large star block copolymer entity which was to constitute the main APCN building block. These giant polymeric building blocks were finally interlinked in a homogeneous manner using tri- and tetra-armed hydrophilic star homopolymers to yield the desired APCNs. These APCNs nanophase separated both in water and in the bulk, exhibiting very large domain sizes, up to 67 and 50 nm in water and in the bulk, respectively.

## 2. Experimental section

### Materials and methods

#### Materials

*n*-Butyl acrylate (BuA, ≥99.0%), *N,N*-dimethylacrylamide (DMAAm, ≥98.5%), diacetone acrylamide (DiAcAAm, 99%), 1,6-hexanediol diacrylate (HDDA, 80%), (2-dodecylthiocarbonothioylthio)-2-methylpropionic acid (DMPA, 97%), 2,2ʹ-azobis(isobutyronitrile) (AIBN, 95%), 2,2-diphenyl-1-picrylhydrazyl hydrate (DPPH, 95%), basic alumina (≥97%), calcium hydride (CaH_2_, 90–95%), 1,4-dioxane (≥99.8%), tetrahydrofuran (THF, ≥99.0%, both reagent and HPLC grade), *n*-hexane (≥96%), *N,N*-dimethylformamide (DMF, 99.8%), ethanol (96%) and deuterated chloroform (CDCl_3_, 99.8%) were purchased from Sigma-Aldrich-Merck, Darmstadt, Germany.

#### Methods

The BuA, DMAAm and DiAcAAm monomers and the HDDA crosslinker were passed through basic alumina columns to remove inhibitors and any other acidic impurities. Then, they were thoroughly dried by being stirred over CaH_2_ for 72 h, in the presence of the DPPH free radical inhibitor which averts undesired thermal polymerization. Finally, they were freshly vacuum-distilled over CaH_2_ just prior to use. The polymerization solvent, 1,4-dioxane, was also dried over CaH_2_, and was freshly vacuum-distilled over CaH_2_ prior to use. The AIBN radical initiator was purified *via* recrystallization (×2) from ethanol.

### RAFT polymerizations

All ‘core-first’ multi-armed star (called ‘nanogels’, according to the nomenclature by the International Union of Pure and Applied Chemistry, IUPAC) ‘homopolymers’ and ‘copolymers’ were prepared using reversible addition-fragmentation chain transfer (RAFT) polymerization in three or four sequential steps. In all cases, the HDDA crosslinker was polymerized first, and DiAcAAm was polymerized last, with the polymerization of either BuA, or DMAAm or both BuA and DMAAm, taking place as second (for the BuA or DMAAm ‘homopolymers’) or second and third (for the amphiphilic ‘copolymers’) polymerizations. As examples, we describe below the experimental procedure followed for the preparation of the BuA hydrophobic star ‘homopolymer’ (quotation marks are used here because, in addition to the main component, polyBuA, the ‘homopolymer’ also contains HDDA crosslinker units and DiAcAAm end-linking units, rendering it, strictly speaking, a terpolymer rather than a homopolymer) and the equimolar BuA-DMAAm amphiphilic star block ‘copolymer’ (similarly, the ‘copolymer’ referred to here is, actually, a quaterpolymer). The livingness of RAFT polymerization was checked, and confirmed, by monitoring the kinetics, analyzed using ^1^H NMR spectroscopy in CDCl_3_, of BuA growth off an HDDA_6_ core. The relevant semi-logarithmic plot of relative conversion *vs*. time, presented in Figure S1 in the Supporting Information, SI, is reasonably linear, despite the complex star polymer architecture being formed, indicating the controlled nature of the polymerization.

#### Preparation of the ‘core-first’ multi-armed star ‘homopolymer’ of BuA

To a dried (at 120 °C) 50 mL Schlenk flask, HDDA (0.2 g, 0.884 mmol), DMPA (0.0537 g, 0.147 mmol), AIBN (4.8 mg, 0.029 mmol) and freshly distilled 1,4-dioxane (4.5 mL) were transferred in this order. After the dissolution of the reagents and homogenization of the resulting solution, three freeze-evacuate-thaw cycles were performed to degas the solution, which was, subsequently, immersed in an oil bath thermostated at 65°C and kept there for 20 h. The solution was then sampled for characterizing the formed multi-functional polyHDDA core in terms of its molar mass and composition characteristics using GPC and ^1^H NMR spectroscopy, respectively, and BuA (6.3 mL, 5.6523 g, 44 mmol) was added to the Schlenk flask together with another 18.0 mL 1,4-dioxane. The RAFT polymerization of BuA was allowed to proceed for 48 h, and a sample was again withdrawn for molar mass and composition analyses. Finally, DiAcAAm (0.2488 g, 1.47 mmol) was added to the Schlenk flask together with another 2.2 mL 1,4-dioxane. The RAFT polymerization of DiAcAAm was left to proceed for 24 h, and samples for GPC and ^1^H NMR spectroscopy were obtained. The HDDA_6_-*b*-BuA_300_-*b*-DiAcAAm_10_ star polymer solution was, subsequently, diluted with THF and precipitated three times in cold *n*-hexane for purification. A similar procedure was followed for the synthesis of the hydrophilic DMAAm star ‘homopolymer’.

#### Preparation of the ‘core-first’ multi-armed equimolar BuA-DMAAm amphiphilic star ‘copolymer’

To a dried (at 120°C) 50 mL Schlenk flask, HDDA (0.2 g, 0.884 mmol), DMPA (0.0537 g, 0.147 mmol), AIBN (4.8 mg, 0.029 mmol) and freshly distilled 1,4-dioxane (4.5 mL) were transferred in this order. The reagents were dissolved in 1,4-dioxane, and the resulting solution was homogenized. After being degassed through three freeze-evacuate-thaw cycles, the solution was immersed in an oil bath thermostated at 65°C and kept there for 20 h. Samples were then withdrawn for molar mass and composition characterization, and BuA (3.15 mL, 2.8262 g, 22 mmol) was added to the Schlenk flask together with another 4.7 mL 1,4-dioxane. BuA was allowed to polymerize for 24 h, and sampling was performed again, followed by the addition of 2.2 mL DMAAm (2.1858, 22 mmol) and 1,4-dioxane (another 4.4 mL). DMAAm was left to polymerize for 48 h and samples were obtained. Finally, DiAcAAm (0.2488 g, 1.47 mmol) was added to the Schlenk flask together with another 2.2 mL 1,4-dioxane. The RAFT polymerization of DiAcAAm was allowed to progress for 24 h, and samples for molar mass and composition determination were taken. The HDDA_6_-*b*-BuA_150_-*b*-DMAAm_150_-*b*-DiAcAAm_10_ amphiphilic star ‘copolymer’ solution was, subsequently, diluted with THF and purified *via* three consecutive precipitations in cold *n*-hexane. A similar procedure was followed for the preparation of the two other BuA-DMAAm amphiphilic star block copolymers of different compositions, *via* the appropriate adjustment of the loadings for the BuA and DMAAm comonomers.

### Star polymer characterization

The star polymers and their precursors were characterized in terms of their molar masses and compositions using gel permeation chromatography (GPC) and proton nuclear magnetic resonance (^1^H NMR) spectroscopy, respectively.

#### Gel permeation chromatography (GPC)

GPC was employed to record the GPC traces and molar mass distributions of the multi-arm star polymers and their precursors. A complete set of GPC traces, concerning the star polymer whose all three samples could be well-analyzed using chromatography, is presented in Figure S2 in the SI. From these distributions, the number-average molar masses, *M*_*n*_, and molar mass dispersities, *Ð* (= *M*_*w*_/*M*_*n*_, where *M*_*w*_ is the weight-average molar mass), were calculated from a calibration curve based on nine near-monodisperse linear poly(methyl methacrylate) molar mass (600–250,000 g mol^−1^) standards. GPC was run on a 1260 Infinity II Agilent (Waldbronn, Germany) liquid chromatograph equipped with a 1260 Infinity II isocratic pump, delivering THF at 1 mL min^−1^, a refractive index detector, and a single PLgel-5-μm mixed-D GPC column from Polymer Laboratories (United Kingdom). An OpenLAB CDS Workstation Software also from Agilent was used for calculations of the molar masses and their dispersities.

#### Proton nuclear magnetic resonance (^1^H NMR) spectroscopy

^1^H NMR spectroscopy was utilized to obtain the ^1^H NMR spectra of the multi-arm star polymers and their precursors to confirm their constitution and to calculate their composition when comprising more than one polymeric components. ^1^H NMR spectroscopy was performed on an Avance Bruker (Germany) 500 MHz NMR spectrometer (Karlsruhe, Germany) equipped with an ultra-shield magnet, using deuterated chloroform (CDCl_3_) as solvent. All recorded ^1^H NMR spectra are provided in the SI, as Figures S3 – S7. Copolymer composition was calculated from the ratio of the normalized areas of the peaks at 4.0 ppm (BuA units) and 2.9 ppm (DMAAm units).

### Formation of the polymer (co)networks

The polymer networks were formed by mixing 35% DMF solutions of the tri- or tetra-PEG crosslinkers with the ‘core-first’ multi-armed star (co)polymers (‘nanogels’) at the stoichiometric ratio of their hydrazide and ketone end-groups. As an example, we provide the details for the procedure followed for the formation of the APCN based on the TetraPEG-5000 and HDDA_6_-*b*-BuA_300_-*b*-DiAcAAm_10_ combination. TetraPEG-5000 (0.0258 g, 20 μeq of PEG arms) and HDDA_6_-*b*-BuA_300_-*b*-DiAcAAm_10_ (0.0772 g, 2.0 μeq of star arms, 20 μeq of DiAcAAm monomer repeating units) were separately dissolved in 0.074 and 0.221 mL DMF, respectively. The two DMF solutions were transferred into a 1.75-mL glass vial where they were vigorously mixed using a vortex mixer and allowed at rest for gel formation. A gel was formed within 24 h, but the crosslinking reaction was allowed to proceed for one whole week. Subsequently, the gel was taken out of the vial by breaking the vial, and was vacuum-dried at room temperature in a vacuum oven for 72 h to constant weight.

### Characterization of the APCNs

The APCNs were characterized in terms of their equilibrium swelling degrees in water, and also in terms of their structure in the bulk and in water using wide- and small-angle X-ray scattering (WAXS and SAXS).

#### Measurements of the degrees of swelling (DS) in water

Vacuum-dried gel pieces were weighed and were then transferred into excess water where they were allowed to equilibrate for one week. Afterward, they were drained with tissue paper and weighed again. The aqueous degree of swelling was calculated as the ratio of the water-swollen divided by the dried gel masses.

#### Wide- and small-angle X-ray scattering (WAXS and SAXS)

The structure of the APCNs in the bulk and in water was characterized using WAXS and SAXS. The measurements were performed using a laboratory GANESHA 300XL instrument (SASXLab ApS, Copenhagen, Denmark) with a wavelength of 0.154 nm. Samples were loaded in sandwich holders and sealed with 5–7 µm thick mica windows. The measurements were carried out at room temperature. The scattered intensity was recorded using a PILATUS 300K detector (pixel size 172 µm) at sample-to-detector distances of 96, 396 and 1046 mm with exposure times of 300, 600 and 1200 s, respectively, to cover a broad range of momentum transfer, *q*. Azimuthally averaged scattering curves at all distances were merged, and the background scattering of an empty sample holder was subtracted.

The SAXS data (up to *q* = 2.0 nm^−1^) were fitted to structural models. For most samples, the hydrophobic nanodomains were modeled as spheres with radii having a Gaussian distribution [22]. Their correlation was described using the Percus-Yevick hard sphere structure factor [23,24], comprising, among others, the hard-sphere radius, *R*_HS_, *i.e*., half the average distance between the centers of the spheres, and the volume fraction of correlated spheres, *η*. These terms were chosen as the simplest model, based on the least number of parameters. This model was sufficient to describe the data from the dry samples, except sample no. 2 (sample notation as in Table 5). For most swollen samples (samples no. 1, 2, 4 and 5), an additional Ornstein-Zernike structure factor was added [25], giving the correlation length of fluctuations of the water-soluble polymer matrix, *ξ*. The SAXS data from the dry sample no. 2 were described by a model for lamellar layers correlated by a Caillé structure factor [26,27], giving, among others, the layer thickness, *δ*, and the repeat distance, *d*. The data from the dry samples no. 5 and 6 were modeled by lamellar layers correlated by a paracrystal structure factor [28]. It comprises, among others, the layer thickness, *δ*, and the repeat distance, *d*. The data from the swollen sample no. 6 were described by a sum of a Porod term to describe the decay at low *q*-values, due to large-scale inhomogeneities, and the Ornstein-Zernike structure factor.

## 3. Results and discussion

### Multi-armed star block copolymer design and synthesis

The aim in this investigation is the fabrication of polymer networks based on star polymers. The use of star polymers as building blocks for the synthesis of polymer networks secures the structural homogeneity of the resulting networks, which, in turn, may lead to the improvement of the network properties, including their mechanical and self-assembling properties. Whereas the first literature reports on the preparation of polymer networks based on star polymers appeared 18–25 years ago [29–32], there is an increasing research activity in this area, manifested by the several publications that appeared within the last 12 months [33–38]. Furthermore, two nice reviews have been published, summarizing the intense activity in this field [39,40].

Preliminary experiments indicated difficulty in obtaining monodisperse star polymers with degrees of polymerization (DP) equal to 20 or higher (a DP of 100 was initially planned) using a tetrafunctional, R-group linked, chain transfer agent (CTA) *via* reversible addition-fragmentation chain transfer (RAFT) polymerization [21]. Thus, we modified our originally planned approach and, instead, we pursued a ‘core-first’ star (‘nanogel’) synthesis technique, using a monofunctional CTA (essentially being a single branch of the above-used tetrafunctional CTA), in four sequential RAFT polymerization addition steps in one-pot, affording the preparation of multi-armed (rather than tetra-armed) end-functionalized star block copolymers. After optimization, this approach allowed us to reach an arm DP of 300. It is noteworthy that we have used in the past the ‘core-first’ approach to synthesize star precursors for APCN preparation both using group transfer polymerization (GTP) [41,42] and RAFT polymerization [43], but the arm DPs then reached values of only 40 and 100, respectively.

**Figure 1.**
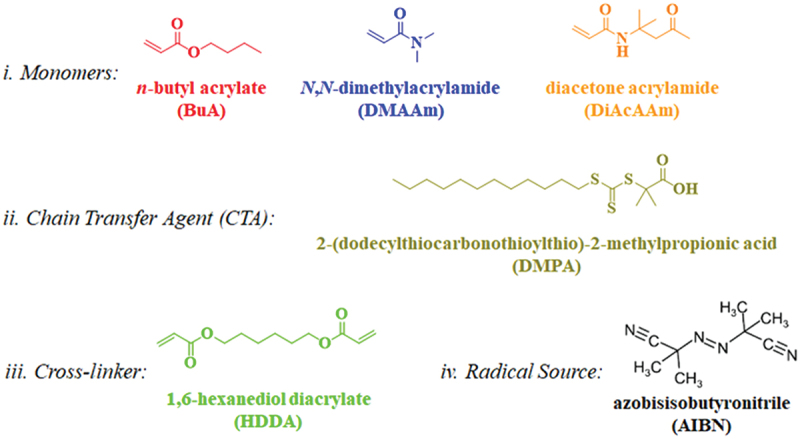
Chemical structures, names and abbreviations of the reagents used for the synthesis of the end-functionalized multi-armed amphiphilic star block copolymers *via* a one-pot, four-step, sequential RAFT polymerization.

The above-mentioned four sequential steps within RAFT polymerization involved the addition of, first, a small amount of divinyl crosslinker that was to form the multifunctional core, was followed by the additions of the hydrophobic and hydrophilic monomers, and was concluded by the addition of a small amount of a ketone-bearing monomer whose functional units would later enable the interconnection among the star polymers to form the APCN. The divinyl crosslinker was hexanediol diacrylate (HDDA), the hydrophobic monomer was *n*-butyl acrylate (BuA), the hydrophilic monomer was *N,N*-dimethylacrylamide (DMAAm), whereas the ketone-functional monomer was diacetone acrylamide (DiAcAAm). The chemical structures, names and abbreviations of the three monomers, the crosslinker, the monofunctional chain transfer agent (CTA), (2-dodecylthiocarbonothioylthio)-2-methylpropionic acid (DMPA), and the radical source, azobisisobutyronitrile (AIBN), are displayed in Figure 1 below.

**Figure 2.**
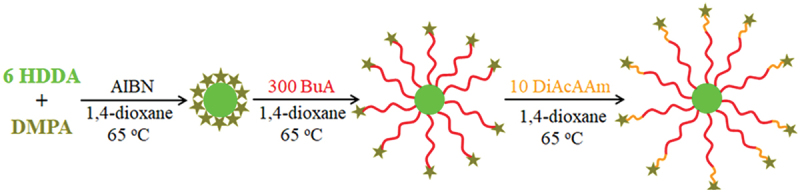
Synthetic sequence yielding the polyBuA multi-armed hydrophobic star ‘homopolymer’, decorated with a short polyketone block at the star’s tip.

In addition to multi-armed amphiphilic star block ‘copolymers’ of various compositions, the two corresponding ‘homopolymers’, hydrophobic and hydrophilic, were also prepared. The synthetic sequence followed for the preparation of the end-functionalized hydrophobic BuA star ‘homopolymer’ is illustrated in Figure 2 presented on the next page.

**Figure 3.**
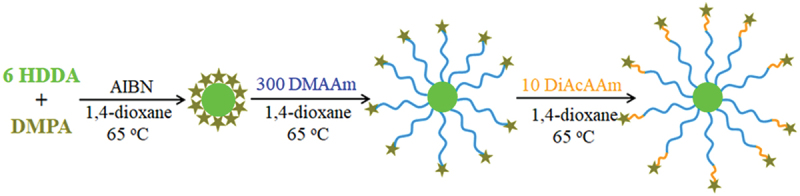
Synthetic sequence resulting in the polyDMAAm multi-armed hydrophilic star ‘homopolymer’, also decorated with a short polyketone end-block.

Similarly, the synthetic sequence followed for the preparation of the end-functionalized hydrophilic DMAAm star ‘homopolymer’ is displayed in Figure 3 also appearing on the next page.

**Figure 4.**
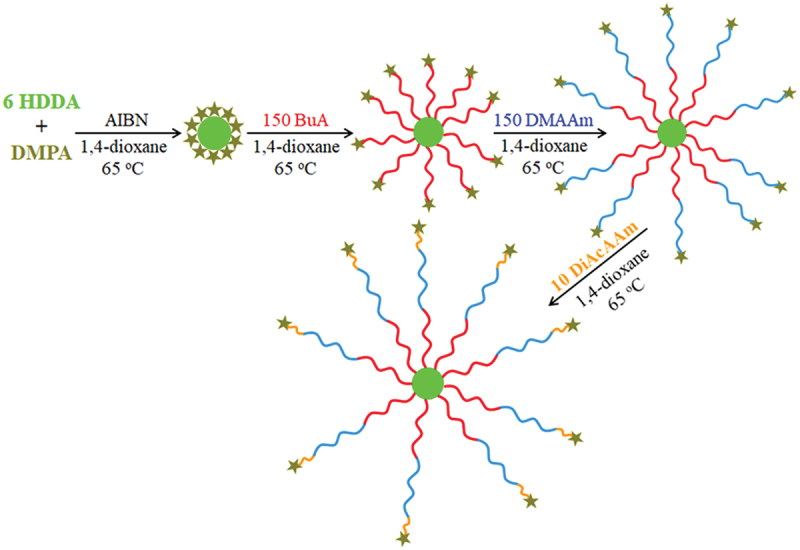
Synthetic sequence followed to provide the polyBuA-polyDMAAm multi-armed equimolar amphiphilic star block ‘copolymer’, bearing a short polyketone terminal block.

Finally, the synthetic sequence followed for the preparation of the end-functionalized equimolar amphiphilic polyBuA-polyDMAAm star block ‘copolymer’ is displayed in Figure 4 shown again on the next page.

In addition to the equimolar amphiphilic polyBuA-polyDMAAm star block ‘copolymer’, two more amphiphilic star block ‘copolymers’ were also prepared, having the same overall arm DP of 300, with the one being BuA-rich and having a structure of HDDA_6_-*b*-BuA_225_-*b*-DMAAm_75_-*b*-DiAcAAm_10_, and the other being DMAAm-rich with a structure of HDDA_6_-*b*-BuA_75_-*b*-DMAAm_225_-*b*-DiAcAAm_10_. The chemical structures of all acrylate-acrylamide star (co)polymers synthesized using RAFT polymerization in this investigation are listed in Table 1 appearing on the next page too.

**Table 1.** Multi-armed star ‘homopolymers’ and ‘copolymers’ prepared *via* a one-pot, sequential RAFT polymerization.

No.	Star Polymer Structure^a^
1	HDDA_6_-*b*-BuA_300_-*b*-DiAcAAm_10_
2	HDDA_6_-*b*-BuA_225_-*b*-DMAAm_75_-*b*-DiAcAAm_10_
3	HDDA_6_-*b*-BuA_150_-*b*-DMAAm_150_-*b*-DiAcAAm_10_
4	HDDA_6_-*b*-BuA_75_-*b*-DMAAm_225_-*b*-DiAcAAm_10_
5	HDDA_6_-*b*-DMAAm_300_-*b*-DiAcAAm_10_

^a^HDDA: 1,6-hexanediol diacrylate crosslinker; BuA: *n*-butyl acrylate; DiAcAAm: diacetone acrylamide; DMAAm: *N,N*-dimethylacrylamide.

### Multi-armed star block ‘copolymer’ characterization

Molar mass and composition information for the acrylamide/acrylate multi-armed star block copolymers was collected using gel permeation chromatography (GPC) and proton nuclear magnetic resonance (^1^H NMR) spectroscopy. The former technique yielded the number-average molar masses, *M*_*n*_, and molar mass dispersities, *M*_*w*_/*M*_*n*_, relative to narrow molar mass distribution linear poly (methyl methacrylate) standards, whereas the latter technique provided copolymer composition and monomer conversion. All this information is assembled and listed in Table 2 in the bottom of this page.

**Table 2.** Molar masses, molar mass distributions and monomer conversions for the multi-armed acrylate-acrylamide star (co)polymers prepared *via* sequential RAFT copolymerization.

No.	Polymer Structure in Each Step^a^	Monomer Conversion by ^1^H NMR	GPC Results	
		Spectroscopy (mol%)	*M*_*n*_ (g mol^−1^)	*M*_*w*_/*M*_*n*_
1	**HDDA**_**6**_**-*b*-BuA**_**300**_**-*b*-DiAcAAm**_**10**_			
1a	HDDA_6_	100	15,500	1.93
1b	HDDA_6_-*b*-BuA_300_	86	632,000	1.23
			204,000	1.07
1c	HDDA_6_-*b*-BuA_300_-*b*-DiAcAAm_10_	89	903,000	1.19
			223,000	1.18
2	**HDDA**_**6**_**-*b*-BuA**_**225**_**-*b*-DMAAm**_**75**_**-*b*-DiAcAAm**_**10**_			
2a	HDDA_6_	100	14,800	1.99
2b	HDDA_6_-*b*-BuA_225_	89	402,000	1.36
			47,900	1.09
2c	HDDA_6_-*b*-BuA_225_-*b*-DMAAm_75_	100	—	—
2d	HDDA_6_-*b*-BuA_225_-*b*-DMAAm_75_-*b*-DiAcAAm_10_	30	—	—
3	**HDDA**_**6**_**-*b*-BuA**_**150**_**-*b*-DMAAm**_**150**_**-*b*-DiAcAAm**_**10**_			
3a	HDDA_6_	100	13,400	2.22
3b	HDDA_6_-*b*-BuA_150_	86	271,000	1.60
			28,500	1.16
3c	HDDA_6_-*b*-BuA_150_-*b*-DMAAm_150_	93	—	—
3d	HDDA_6_-*b*-BuA_150_-*b*-DMAAm_150_-*b*-DiAcAAm_10_	100	—	—
4	**HDDA**_**6**_**-*b*-BuA**_**75**_**-*b*-DMAAm**_**225**_**-*b*-DiAcAAm**_**10**_			
4a	HDDA_6_	100	15,900	2.11
4b	HDDA_6_-*b*-BuA_75_	94	189,000	1.51
			11,600	1.04
4c	HDDA_6_-*b*-BuA_75_-*b*-DMAAm_225_	100	—	—
4d	HDDA_6_-*b*-BuA_75_-*b*-DMAAm_225_-*b*-DiAcAAm_10_	93	—	—
5	**HDDA**_**6**_**-*b*-DMAAm**_**300**_**-*b*-DiAcAAm**_**10**_			
5a	HDDA_6_	100	14,300	2.42
5b	HDDA_6_-*b*-DMAAm_300_	100	—	—
5c	HDDA_6_-*b*-DMAAm_300_-*b*-DiAcAAm_10_	100	—	—

^**a**^HDDA: 1,6-hexanediol diacrylate crosslinker; BuA: *n*-butyl acrylate; DiAcAAm: diacetone acrylamide; DMAAm: *N,N*-dimethylacrylamide.

The DMAAm-containing star polymers could not be analyzed by GPC, as they were undesirably retained onto the GPC column, which prevented their elution and, consequently, the determination of their molar masses. Nonetheless, as all five (final) star polymers have the same arm degree of polymerization (DP), namely 310, and the molar masses of the main two monomers, BuA and DMAAm are rather similar, 128 and 99 g mol^−1^, respectively, the molar masses of the four DMAAm-containing final star polymers should be close to that of the BuA ‘homopolymer’-based star polymer, with a main GPC peak of around 630 000 g mol^−1^. Taking into account the arm (300 BuA units) molar mass of 40,120 g mol^−1^ (this also includes the molar masses of the 6 units of the HDDA crosslinker plus that of the CTA), the molar mass of 632 000 g mol^−1^ corresponds to an average of 16 arms. Upon the addition of the ~10 DiAcAAm units at the periphery of each arm, the overall molar mass increases to just above 900 000 g mol^−1^, corresponding now to 22 arms, representing a slight increase from 16, possibly indicating partial inter-star coupling. This star coupling, in turn, may be due to the linking of free radicals at the tips of two adjacent arms belonging to two different star polymers. However, this linking cannot be extensive, because the trithiocarbonate species exists mostly in its inactive, non-radical form. It is noteworthy that the core (HDDA_6_) molar mass for all samples, determined to be around 15,000 g mol^−1^, corresponds to about 9 arms (single arm molar mass is 1720 g mol^−1^). As mentioned above, these 9 arms later become 16, which further grow to 22, as the BuA and DiAcAAm units are added to the arms, something apparently also accompanied by star-star coupling, facilitated by the high solids concentration in the polymerization flask, employed to increase the ‘livingness’ of the polymerization reaction and attain the rather high targeted DPs.

As the arm DP of BuA increases from 75 to 300 in the BuA star homopolymers, the molar masses expectedly increase from 189 000 to 632 000 g mol^−1^, while the arm numbers vary in the range between 13 and 17. It is noteworthy that the molar mass distributions of the BuA star homopolymers are tri- or bi-modal, bearing, in addition to the main star peak, a second peak corresponding to single, unattached arms, or stars with a lower arm number, up to 5.

The column in Table 2 labeled monomer conversion by ^1^H NMR spectroscopy records the conversion of the monomer (BuA, DMAAm or DiAcAAm) or crosslinker (HDDA) added first. Except for one case, all conversions are quantitative (100%) or near-quantitative (86–94%), indicating that all species introduced in the polymerization reactor were practically incorporated in the star polymer. The one case where conversion was insufficient, at 30%, concerns the DiAcAAm ‘sticking’ units in star block copolymer no. 2, with structure, HDDA_6_-*b*-BuA_225_-*b*-DMAAm_75_-*b*-DiAcAAm_10_. This means that the peripheral polyDiAcAAm block contains only 3 rather than 10 units. Note that, although only one DiAcAAm ‘sticking’ unit per star arm would suffice to later attain star interconnection and gel formation, the incorporation of 10 DiAcAAm units was targeted so as to render gelation easier. According to ^1^H NMR spectroscopic analysis, except for sample no. 2, the other four star samples carried 9 or 10 DiAcAAm units per arm. Thus, arm content in DiAcAAm in most stars is between 9 and 10 units.

### Three- and four-armed poly(ethylene glycol) star crosslinkers

The above ketone-bearing, multi-armed star (co-)polymers were crosslinked to form polymer (co)networks using benzaacylhydrazide-end-functionalized three- or four-armed poly(ethylene glycol) (PEG) star homopolymers (triPEG and tetraPEG stars). Although more complicated than our previously-reported *in situ* crosslinking procedure using divinyl compounds [41–43], the present crosslinking method leads to networks with improved structure because of the precisely-known functionalities of the triPEG and tetraPEG star crosslinkers. These triPEG and tetraPEG stars with their three or four termini end-functionalized with benzaacylhydrazide groups were obtained from our previous works, and their molar masses are displayed in Table 3 below [44,45].

**Table 3.** Three- [44] and four-armed [45] star PEG homopolymers with benzaacylhydrazide terminal groups used as crosslinkers for APCN formation.

No.	Star Polymer Name	Core Functionality	Molar Mass (g mol^−1^)
1	TriPEG-1000	3	1000
2	TetraPEG-5000	4	5000
3	TetraPEG-10000	4	10,000
4	TetraPEG-20000	4	20,000

### Gel formation

The ketone-end-functionalized acrylamide/acrylate star (block co)polymers were combined with the benzaacylhydrazide PEG star crosslinkers to form the APCNs. To this end, 35% w/w solutions of each of the two reactants were prepared in a common, non-selective solvent, *N,N*-dimethylformamide (DMF), and at the stoichiometric ratio of the two types of the reactive end-groups. The solutions were combined, vigorously vortex-mixed, and allowed to react for  one week. Table 4 lists the combinations of the acrylamide/acrylate star (block co)polymers with the PEG star crosslinker explored for gel formation.

**Table 4.** Gels formed by the combination of the acrylamide/acrylate star (block co)polymers with the star PEG crosslinkers.

No.	Amphiphilic Polymer Conetwork Components^a^	Gel Formation?
1	HDDA_6_-*b*-BuA_300_-*b*-DiAcAAm_10_ – TriPEG-1000	Yes
2	HDDA_6_-*b*-BuA_300_-*b*-DiAcAAm_10_ – TetraPEG-5000	Yes
3	HDDA_6_-*b*-BuA_300_-*b*-DiAcAAm_10_ – TetraPEG-10000	Yes
4	HDDA_6_-*b*-BuA_300_-*b*-DiAcAAm_10_ – TetraPEG-20000	No
5	HDDA_6_-*b*-BuA_150_-*b*-DMAAm_150_-*b*-DiAcAAm_10_ – TriPEG-1000	Yes
6	HDDA_6_-*b*-BuA_150_-*b*-DMAAm_150_-*b*-DiAcAAm_10_ – TetraPEG-5000	Yes
7	HDDA_6_-*b*-BuA_150_-*b*-DMAAm_150_-*b*-DiAcAAm_10_ – TetraPEG-10000	Yes
8	HDDA_6_-*b*-BuA_150_-*b*-DMAAm_150_-*b*-DiAcAAm_10_ – TetraPEG-20000	Yes
9	HDDA_6_-*b*-BuA_75_-*b*-DMAAm_225_-*b*-DiAcAAm_10_ – TriPEG-1000	Yes
10	HDDA_6_-*b*-BuA_75_-*b*-DMAAm_225_-*b*-DiAcAAm_10_ – TetraPEG-5000	Yes
11	HDDA_6_-*b*-DMAAm_300_-*b*-DiAcAAm_10_ – TriPEG-1000	Yes
12	HDDA_6_-*b*-DMAAm_300_-*b*-DiAcAAm_10_ – TetraPEG-5000	Yes

^a^HDDA: 1,6-hexanediol diacrylate crosslinker; BuA: *n*-butyl acrylate; DiAcAAm: diacetone acrylamide; DMAAm: *N,N*-dimethylacrylamide; triPEG-1000: trihydrazide end-functionalized three-armed poly(ethylene glycol) (PEG) star polymer of 1000 g mol^−1^ molar mass; tetraPEG-MM: tetrahydrazide end-functionalized four-armed poly(ethylene glycol) (PEG) star polymers of different molar masses (MM): 5000, 10,000 or 20,000 g mol^−1^.

The table shows that, except for one case, all examined combinations resulted in gel formation. All gel formation times were shorter than 24 h. The combination that did not give a gel within a week (total observation time) from the time of mixing the two components was the polyBuA star homopolymer with the largest tetraPEG crosslinker (20000 g mol^−1^). In this case, the concentration of the hydrazide reacting end-groups was lowest, which would render the crosslink formation reaction very slow. Furthermore, even small deviations from the targeted perfect stoichiometry in this case could make gel formation too slow, even impossible.

### Mechanical properties in the bulk and swelling in water

The APCN gels formed in DMF were vacuum dried to remove the synthesis solvent. The obtained dried (bulk) samples had rather poor mechanical properties, and could not be tested in tension. However, a qualitative description of the mechanical features of these samples in the bulk, and their equilibrium degrees of swelling in aqueous phosphate buffer (100 mM) at pH = 7.2 are given in Table 5 on the next page. The last column in the table shows that, in their bulk (dried) state, the star block copolymer-based samples (samples no. 3–6, please note that sample numbering in Table 5 is different from Table 4) were rather stiff and fragile, a result of their containing the glassy polyDMAAm segments (polyDMAAm has a *T*_*g*_, of + 89°C [46]) in the star arms. The aqueous swelling degrees of the same samples displayed moderate values, in the range between 3.3 and 5.8, a result of their fair polyDMAAm content, and increased with the size of the hydrophilic crosslinker, from the marginally hydrophilic triPEG-1000 crosslinker to the more hydrophilic tetraPEG ones with overall molar masses from 5000 to 20,000 g mol^−1^. On the other hand, samples no. 1 and 2, which were those based on the hydrophobic polyBuA star ‘homopolymer’, crosslinked together with the two smallest tetraPEG crosslinkers, tetraPEG-5000 and tetraPEG-10000, exhibited better mechanical properties, as they were rather soft, a result of the rubbery nature of polyBuA (*T*_*g*_ of −54°C [46]), but they displayed very low aqueous swelling degrees, 1.3 and 1.5, arising from their increased hydrophobicity.

**Table 5.** Chemical composition of the star polymer-based APCNs, their aqueous degrees of swelling and their qualitative compressive mechanical properties in the dried state.

No.^a^	Amphiphilic Polymer Conetwork Components^b^	Aqueous Swelling Degree	Sample Texture in the Bulk
1	HDDA_6_-*b*-BuA_300_-*b*-DiAcAAm_10_ – TetraPEG-5000	1.3	soft
2	HDDA_6_-*b*-BuA_300_-*b*-DiAcAAm_10_ – TetraPEG-10000	1.5	soft and collapsed
3	HDDA_6_-*b*-BuA_150_-*b*-DMAAm_150_-*b*-DiAcAAm_10_ – TriPEG-1000	3.3	stiff
4	HDDA_6_-*b*-BuA_150_-*b*-DMAAm_150_-*b*-DiAcAAm_10_ – TetraPEG-5000	4.0	stiff
5	HDDA_6_-*b*-BuA_150_-*b*-DMAAm_150_-*b*-DiAcAAm_10_ – TetraPEG-10000	4.2	stiff
6	HDDA_6_-*b*-BuA_150_-*b*-DMAAm_150_-*b*-DiAcAAm_10_ – TetraPEG-20000	5.8	stiff

^a^Renumbered, different numbers relative to Table 4.

^b^HDDA: 1,6-hexanediol diacrylate crosslinker; BuA: *n*-butyl acrylate; DiAcAAm: diacetone acrylamide; DMAAm: *N,N*-dimethylacrylamide; TriPEG-1000: trihydrazide end-functionalized three-armed poly(ethylene glycol) (PEG) star polymer of 1000 g mol^−1^ molar mass; tetraPEG-MM: tetrahydrazide end-functionalized four-armed poly(ethylene glycol) (PEG) star polymers of different molar masses (MM): 5000, 10,000 or 20,000 g mol^−1^.

### APCN self-organization

**Figure 5.**
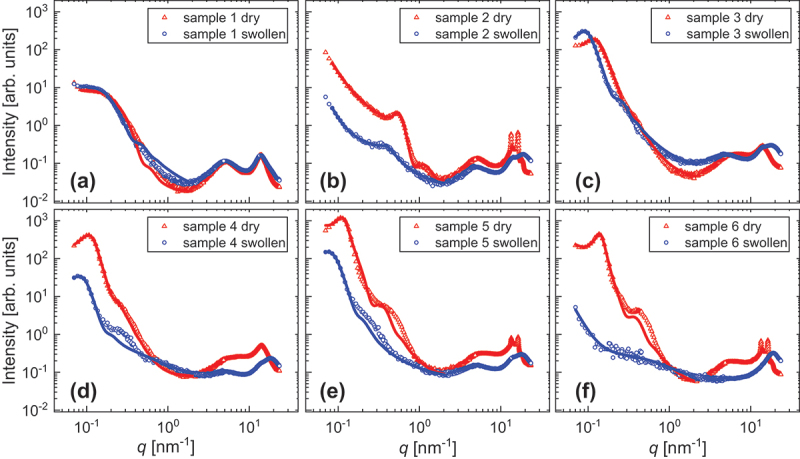
Small- and wide-angle X-ray scattering (SAXS and WAXS) profiles at room temperature for tri- and tetra-PEG crosslinked star APCNs in water (blue symbols) and in the bulk (red symbols). Sample numbering is according to Table 5. The lines are model fits to the data in the SAXS region (see text).

Vacuum dried (bulk) APCNs were examined in terms of structure formation (nanophase separation) using small- and wide-angle X-ray scattering (SAXS and WAXS) at room temperature. Following this characterization, water (H_2_O) was added to the same samples, and the characterization was repeated in the equilibrium H_2_O-swollen state again at room temperature. All recorded SAXS/WAXS intensity profiles are illustrated in Figure 5, where the profiles of a certain sample in the bulk and H_2_O-swollen states are overlaid in the same graph. Each graph also contains appropriate model fits within the SAXS range both for the dried and water-equilibrated samples. From these fits, the characteristic distances were calculated, they are listed in Table 6 and are discussed in detail at the end of the manuscript. Sample numbering in Figure 5 is according to that in Table 5.

**Table 6.** Morphologies and repeat distances of the star polymer-based APCNs in the bulk and equilibrium-swollen in H_2._O.

No.	Amphiphilic Polymer Conetwork Components^a^	Bulk		Water	
		Morphology	Repeat Distance (nm)	Morphology	Repeat Distance (nm)
1	HDDA_6_-*b*-BuA_300_-*b*-DiAcAAm_10_ – TetraPEG-5000	spheres	32^b^	spheres	34^b^
2	HDDA_6_-*b*-BuA_300_-*b*-DiAcAAm_10_ – TetraPEG-10000	lamellae	10^c^	spheres	34^b^
3	HDDA_6_-*b*-BuA_150_-*b*-DMAAm_150_-*b*-DiAcAAm_10_ – TetraPEG-1000	spheres	47^b^	spheres	62^b^
4	HDDA_6_-*b*-BuA_150_-*b*-DMAAm_150_-*b*-DiAcAAm_10_ – TetraPEG-5000	spheres	53^b^	spheres	64^b^
5	HDDA_6_-*b*-BuA_150_-*b*-DMAAm_150_-*b*-DiAcAAm_10_ – TetraPEG-10000	lamellae	50^c^	spheres	67^b^
6	HDDA_6_-*b*-BuA_150_-*b*-DMAAm_150_-*b*-DiAcAAm_10_ – TetraPEG-20000	lamellae	42^c^	—–	—–

^a^HDDA: 1,6-hexanediol diacrylate crosslinker; BuA: *n*-butyl acrylate; DiAcAAm: diacetone acrylamide; DMAAm: *N,N*-dimethylacrylamide; TriPEG-1000: trihydrazide end-functionalized three-armed poly(ethylene glycol) (PEG) star polymer of 1000 g mol^−1^ molar mass; tetraPEG-MM: tetrahydrazide end-functionalized four-armed poly(ethylene glycol) (PEG) star polymers of different molar masses (MM): 5000, 10,000 or 20,000 g mol^−1^.

^b^Sphere-to-sphere repeat distance = 2 × *R*_HS_.

^c^Lamellar inter-layer distance.

The SAXS profile of the most hydrophobic sample, sample no. 1 (Figure 5(a)), based on the hydrophobic polyBuA star ‘homopolymer’ interconnected with the rather small hydrophilic tetraPEG-5000 crosslinker, when in the dried state, exhibits a weak maximum at *q* = 0.01 – 0.2 nm^−1^. The model fit reveals that the hydrophobic nanodomains have an average center-to-center distance of 2 × *R*_HS_ = 32 nm, with a volume fraction of correlated spheres *η* = 0.04, which is a low value. Thus, the hydrophobic peaks are weakly correlated, which may be attributed to the large volume fraction of the hydrophobic component (0.75) and the rather low incompatibility between polyBuA and PEG. (Since the fit is not perfect at *q*-values above 0.3 nm^−1^, we refrain from discussing the sphere radius.) In the WAXS range, two broad maxima are present at 5 and 14 nm^−1^ (in the bulk state), which may reflect local correlations, *e.g*., between the polyBuA chains. There is no sign of crystalline order, though, as expected from the chemical composition of this sample.

Swelling this sample in water causes only a slight shift of the weak maximum in the SAXS range to lower *q*-values, and nearly no changes in the WAXS range. Modeling reveals that the distance between the hydrophobic nanodomains has increased to 34 nm, while *η* has slightly increased to 0.08, *i.e*., the correlation is slightly stronger than in the absence of water, possibly due to the relaxation of stress during the swelling. The fact that these changes are only weak may be related to the very low degree of swelling of this sample in water (=1.3, Table 5), arising from the large incompatibility between polyBuA and water.

Also based on the hydrophobic polyBuA star ‘homopolymer’, but interconnected with the more hydrophilic tetraPEG-10000 crosslinker, the SAXS data of dry sample no. 2 do present two strong correlation peaks in the SAXS range, namely a strong one at 0.5 nm^−1^ and a weaker one at 1.1 nm^−1^ (Figure 5(b)). The factor of *ca*. 2 between these positions points to a nanophase-separated, lamellar morphology. Indeed, the data can be modeled by a lamellar stack with a layer thickness *δ* = 7.3 nm and a repeat distance *d* = 10 nm. In the WAXS range, in addition to a broad maximum at 5 nm^−1^, sharp peaks are present at *q* = 13.4 and 16.4 nm^−1^. These positions are close to the ones expected for the (120) and (032) reflections from crystalline poly(ethylene oxide), namely 13.6 and 16.5 nm^−1^, respectively [47,48]. Thus, the dry sample no. 2 features crystalline PEG, and we speculate that the peak at 0.5 nm^−1^ is due to the formation of crystalline PEG lamellae that alternate with amorphous polyBuA lamellae. A lamellar morphology would be consistent with the close-to-symmetric composition (volume fraction of the hydrophobic component is 0.60), and also the ‘stiff and collapsed’ texture of this sample in the bulk, as was qualitatively described in Table 5.

Upon swelling sample no. 2 in water, the sharp peak in the SAXS regime vanishes and is replaced by a broad correlation peak at 0.5 nm^−1^. Moreover, the diffraction peaks of crystalline PEG in the WAXS regime vanish. The SAXS data can be modeled by correlated spheres having an average distance of 34 nm and a low degree of correlation (*η* = 0.08). We attribute these changes to the swelling of the PEG blocks and the change from a crystallization-induced morphology in the dry state to a gel composed of hydrophobic nanodomains connected by swollen PEG blocks.

The dry samples no. 3 and 4 both feature a maximum at 0.11 nm^−1^, an additional shoulder at 0.2–0.5 nm^−1^, as well as broad maxima in the WAXS range (Figure 5(c) and (d)). In these samples, the PEG blocks are presumably too short or too constrained to crystallize, and the nanophase-separated structure is due to the demixing of polyBuA and PEG. The model fits reveal spheres having radii of 14 and 16 nm, with average center-to-center distances of 47 and 53 nm, and volume fractions of correlated micelles *η* of 0.23 and 0.28, respectively for dry samples no. 3 and 4.

In their water-swollen state, samples no. 3 and 4 exhibit the same features as in their dry state, albeit the ones in the SAXS range are shifted to lower *q*-values, due to swelling. Fitting the same model of correlated spheres reveals that the center-to-center distances have increased to 62 and 64 nm, respectively, while the *η*-values have not changed significantly. We conclude that the morphology has not changed qualitatively by swelling, presumably because in both samples, the PEG blocks are short (1000 and 5000 g mol^−1^) and cannot swell significantly.

In the data from sample no. 5 in the dry state, the reflections from crystalline PEO are present in the WAXS range, as well as a pronounced peak and a shoulder in the SAXS range at 0.11 and 0.40 nm^−1^, respectively (Figure 5(e)). Similar features are observed in both the WAXS and SAXS data from the dry sample no. 6 (Figure 5(f)) with the peaks in the SAXS range being located at higher values, namely 0.14 and 0.41 nm^−1^ (Figure 5(f)). The SAXS data could be modeled by a stack of layers with repeat distances *d* of 50 and 42 nm for dry samples no. 5 and 6, respectively. It is interesting that, for sample no. 6, the repeat distance is smaller than for sample no. 5, despite the higher PEG crosslinker molar mass in sample no. 6. It seems that the latter does not play a major role, since the lamellar structure is most probably caused by crystalline PEG layers. The more pronounced secondary peak in the SAXS data from dry sample no. 6 points to a stronger effect of the crystallization of PEG-20000 than the one of PEG-10000 in dry sample no. 5.

Upon swelling sample no. 5 in water, the features in the SAXS range are still present, albeit shifted to lower *q*-values, while the Bragg reflections from crystalline PEG have vanished. The SAXS data can be modeled by spherical nanodomains that feature an average center-to-center distance of 67 nm and a rather strong degree of correlation (*η* = 0.21). We speculate that now, the demixing of hydrophobic and hydrophilic blocks is at the origin of nanophase separation, and not the crystallization.

The reflections from crystalline PEO are also absent in the WAXS data from the water-swollen sample no. 6, and the SAXS data are quite featureless. Instead of the nanophase-separated structure, only a decay at low *q*-values (up to *ca*. 0.2 nm^−1^), which we attribute to large-scale inhomogeneities in the swollen network, and a decay up to *ca*. 1 nm^−1^ are present. The latter is reminiscent of a simple homopolymer network, and from the fit of the Ornstein-Zernike structure factor, the correlation length *ξ* is estimated at *ca*. 2 nm. Apparently, the long PEG chains dominate the scattering, and the polyBuA blocks do not nanophase-separate any longer from the water-soluble matrix.

We conclude that the morphology of the dry samples depends largely on the length of the polyBuA blocks in the stars, and networks feature correlated hydrophobic nanodomains, but also lamellar structures have been observed, which are induced by the crystallization of the PEG blocks, which seems possible when they are sufficiently long. The water-swollen samples still feature nanophase-separation, except the sample with the lowest hydrophobic content. The nanophase-separated structures in water are spheres in all cases.

Table 6 summarizes the above-determined morphologies of the six samples in the bulk and in water, and the corresponding repeat distances. The repeat distances for spheres in the table were calculated as twice the value of the hard-sphere radius, *R*_HS_, obtained from the model fit to spheres, whereas those for lamellae were the inter-layer spacings obtained from the fit to lamellae. These repeat distances ranged between 10 and 53 nm for the samples in the bulk, whereas the repeat distances for the same samples in water were between 34 and 67 nm. These repeat distances are larger than the corresponding values of 10–26 nm exhibited by water-swollen APCNs also based on interconnected ‘core-first’ star polymers [41–43], and of 5.5–40 nm reported in other published APCN literature [49–65].

In this section, we compare the repeat distances determined from the model fits to the SAXS data with the molecular dimensions of the main APCN component, *i.e*., the multi-armed star polymers. We make this comparison in order to show that the structural APCN spacings are highly related to the star size, and strengthen our design concept that larger star polymers result in larger APCN nanodomains. Regarding the molecular dimensions of the multi-armed star polymers, we estimated the upper and lower limit of their diameter. The upper limit is reached when the arms are fully stretched to their contour length, while the lower limit is attained when the star arms are fully collapsed and contain no solvent. For simplicity, these calculations were restricted only to a BuA_300_ star homopolymer, having a molar mass of 903 000 g mol^−1^ as determined using GPC and shown in Table 2.

Using a contribution of 0.254 nm per acrylate monomer repeating unit to the contour length, this gives a contour length of 76.2 nm (=300 × 0.254 nm) for the BuA_300_ arm, and 152.4 nm (=2 × 76.2 nm) for the star when all of its arms are fully stretched. At the other extreme, for a fully-collapsed BuA_300_ multi-armed star with a molar mass of 903 000 g mol^−1^, and taking into account the polyBuA density of 1.087 g cm^−3^, we calculated a star molar volume equal to 830 700 cm^3^ mol^−1^ (=903 000 / 1.087 cm^3^ mol^−1^), which corresponds to a molecular volume equal to 1379 nm^3^ (=830 700 cm^3^ mol^−1^/ Avogadro’s number). Since SAXS data model fitting indicated two possible morphologies, spheres and lamellae, we then went on to estimate how much this star molecular volume would contribute to the spacing in each of the two cases. Regarding spheres, the contribution would be 13.8 nm to the sphere’s diameter, whereas for lamellae, and simplistically assuming a cubic arrangement, the contribution would be 11.1 nm to the cube’s edge. Thus, the above-estimated star molecular dimensions provide the limits within which the structural spacings should lie. These limits are from 11 or 14 nm all the way to 152 nm. Indeed, all spacings from SAXS essentially lie within these two limits, both for the bulk (10–53 nm) and in water (34–67 nm). The fact that the smallest lamellar spacing in the bulk calculated from SAXS at 10 nm is very close to (and slightly lower than) the lower molecular dimension of 11 nm may indeed indicate that the polyBuA arms are fully collapsed within the star, as expected for the bulk APCN state.

In this final section, we consider samples no. 2 and no. 5 in Table 6, both exhibiting lamellae in the bulk, and, upon hydration, they both transform into spheres. We first discuss the enthalpy of the transformation, and, subsequently, speculate on the mechanism of the transition. The melting of the PEG crystallites in the bulk phase is endothermic [66,67], and the necessary enthalpy may be provided by the exothermic PEG hydration [68]. Regarding the transition mechanism, it is very unlikely that the lamellae are transformed directly to spheres during PEG hydration. On the contrary, as PEG hydration proceeds, the lamellae are probably first transformed to some intermediate morphologies, either stable such as worms/cylinders [69,70] or/and metastable such as perforated lamellae [71], before they are finally converted to spheres.

## 4. Conclusions

Herein, we have prepared novel APCNs *via* the combination of two types of star polymers, with the termini of each star type being reactive to those of the other. The first star type comprised very large multi-armed star polymers, either hydrophobic or amphiphilic, of molar masses approaching 1 MDa, while the other comprised smaller hydrophilic three- or four-armed stars of molar masses in the range from 1 to 20 kDa. The aqueous swelling degrees of the APCNs displayed rather low values, from 1.3 to 5.8, a result of their increased hydrophobicity. The bulk APCNs whose main building block was the multi-armed star ‘homopolymer’ of the low-*T*_*g*_ polyBuA were soft, whereas the bulk APCNs whose main building block was the multi-armed amphiphilic equimolar BuA-DMAAm star ‘copolymer’ were stiff due to the relatively high content (50 mol%) of the glassy polyDMAAm. The APCNs exhibited phase separation on the nanoscale, both in the bulk and in water. Lamellar phases were exhibited by the dried APCNs interconnected with the two tetraPEG stars of the highest molar masses, 10 and 20 kDa, with lamellar repeat distances ranging between 10 and 50 nm. The rest of the dried APCNs and most of the water-swollen APCNs were organized into spheres separated at distances of 32–67 nm. These particularly long domain spacings in the bulk and in water constitute record values in the APCN field, and originate from the large size of the multi-armed star polymers used as the main APCN building blocks. Future work could broaden the scope of this study, by employing different hydrophilic and hydrophobic monomers in order to improve the properties of these materials. For example, the use of the more-hydrogen-bonding hydrophobic monomer *n*-butyl acrylamide to replace *n*-butyl acrylate may improve the mechanical properties of the APCNs. Furthermore, utilizing the less hydrophilic and thermoresponsive *N*-isopropylacrylamide in lieu of the hydrophilic *N,N*-dimethylacrylamide may confer a better quality in APCN nanophase separation, which may also be rendered tunable through temperature.

## Supplementary Material

Supplemental Material
