# Supplementary material for: Amphiphilic polymer conetworks based on triPEG and tetraPEG end-linked multi-armed star block copolymers
Source: Sci Technol Adv Mater. 2026 Apr 16;27(1):2658326. doi: 10.1080/14686996.2026.2658326 (PMC13482925; doi:10.1080/14686996.2026.2658326)
Supplement: Supplementary file 1 [file TSTA_A_2658326_SM5766.pdf]

Supporting Information

Amphiphilic Polymer Conetworks Based on TriPEG and TetraPEG End-linked Multi-armed Star Block Copolymers

Constantina K. Varnava, Demetris E. Apostolides, Costas S. Patrickios, Takamasa Sakai, Florian A. Jung, Christine M. Papadakis*

C. K. Varnava, D. E. Apostolides, C. S. Patrickios
Department of Chemistry, University of Cyprus, P. O. Box 20537, 1678 Nicosia, Cyprus.
E-mail: costasp@ucy.ac.cy

T. Sakai
Department of Bioengineering, Graduate School of Engineering, The University of Tokyo, 7-3-1 Hongo, Bunkyo-ku, Tokyo 113-8656, Japan.

F. A. Jung, C. M. Papadakis
TUM School of Natural Sciences, Physics Department, Soft Matter Physics Group, Technical University of Munich, James-Franck-Str. 1, 85748 Garching, Germany.

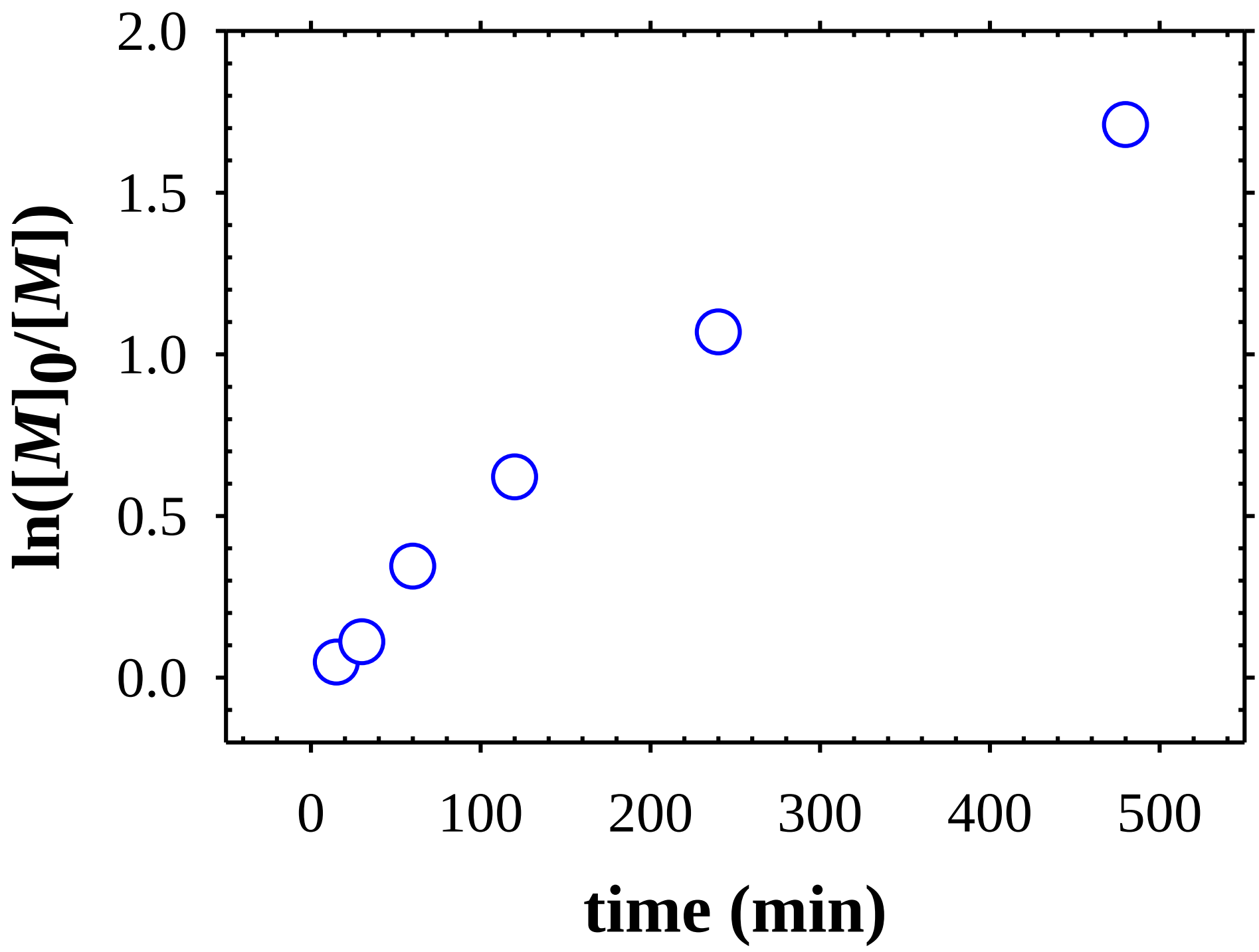

Figure S1. Kinetic study of BuA RAFT polymerization off the ends of an $HDDA_6$ core. The fair linearity of the plot is indicative of the "livingness" of the polymerization.

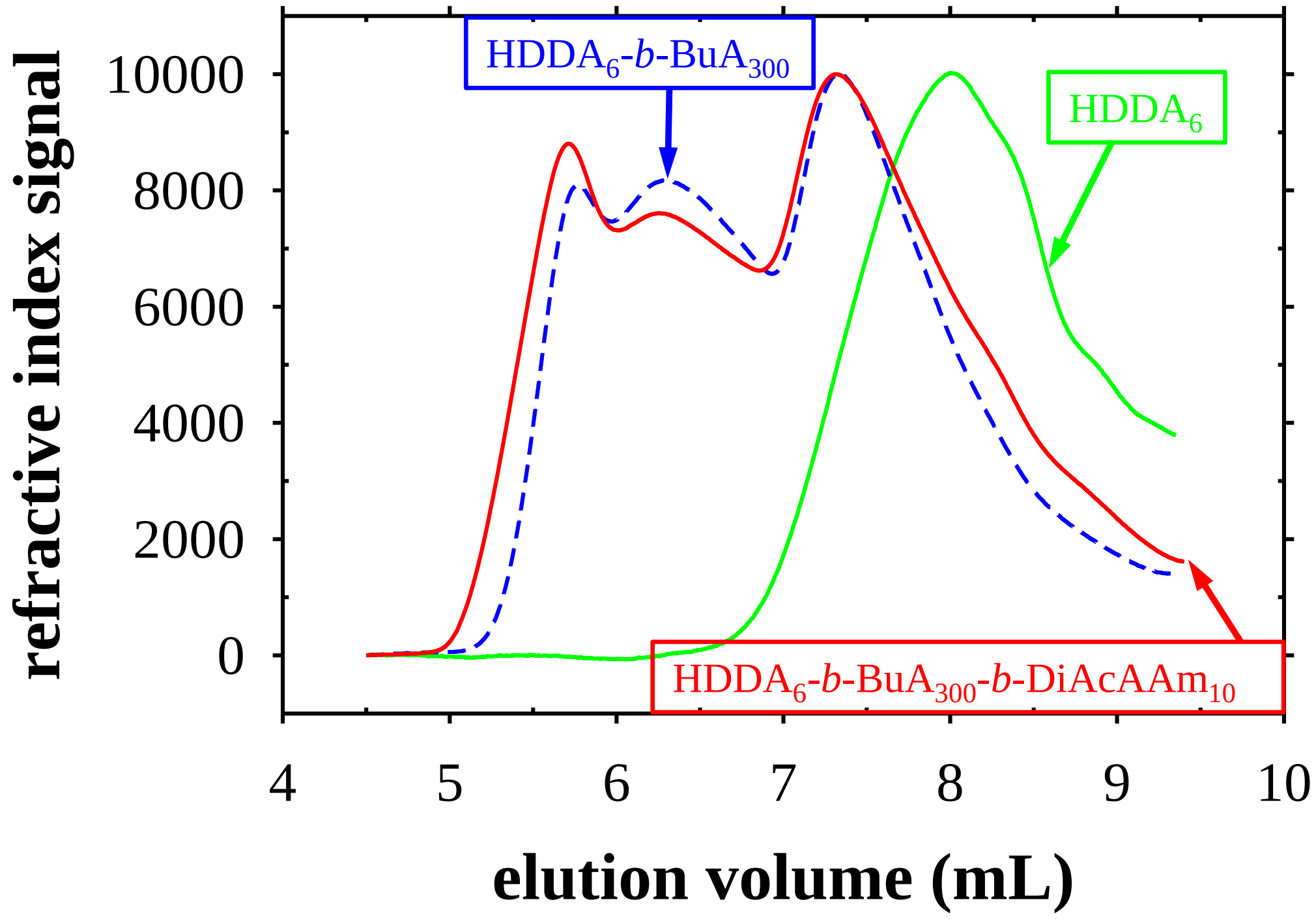

Figure S2. GPC traces of the hydrophobic BuA star "homopolymer", $HDDA_6$-*b*-BuA_{300}-*b*-$DiAcAAm_{10}$, overlaid together with its two precursors, $HDDA_6$ and $HDDA_6$-*b*-BuA_{300}.

Star No. 1

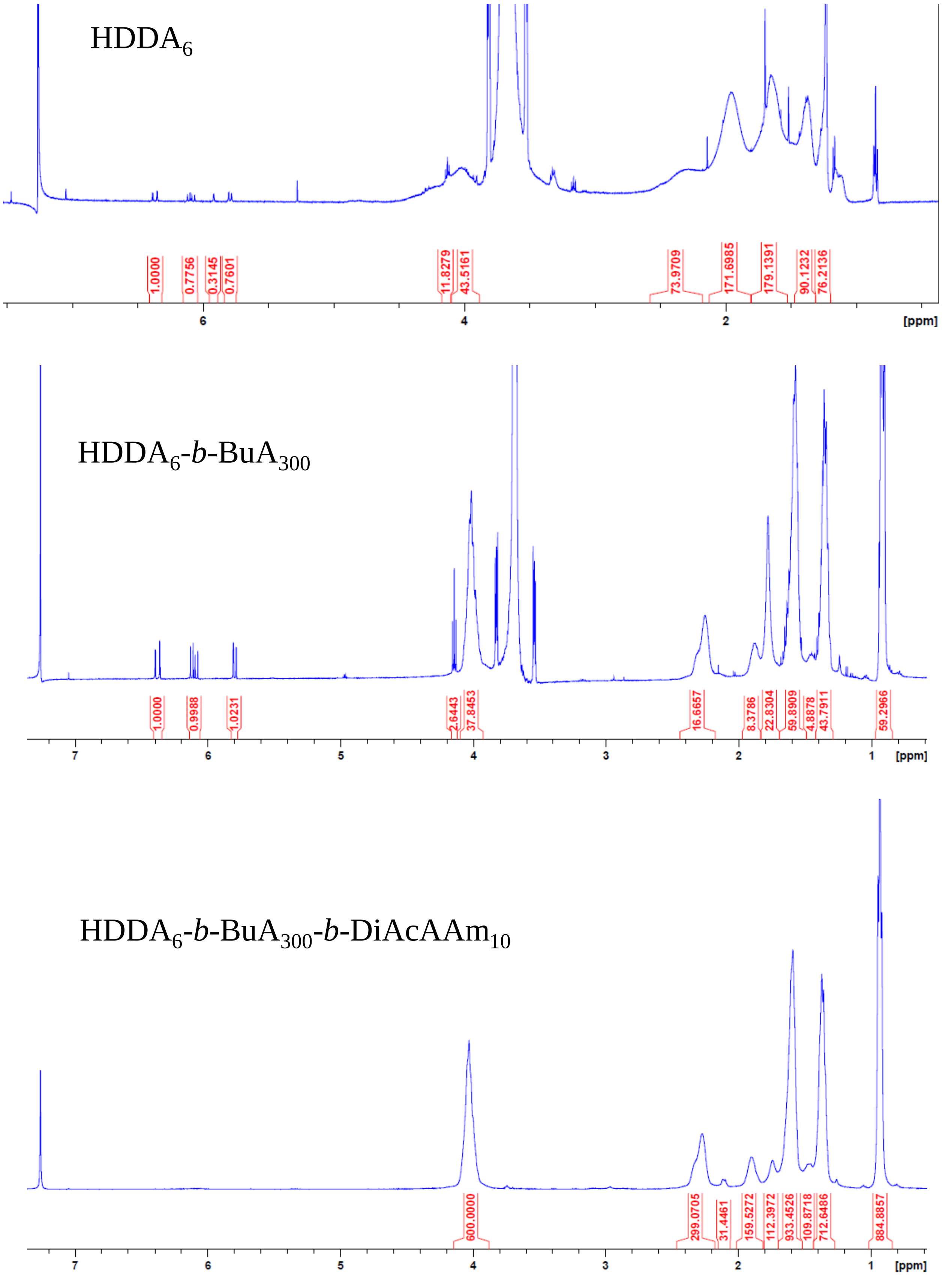

Figure S3. ^{1}H NMR spectra in $CDCl_3$ of the BuA hydrophobic star "homopolymer", HDDA$_6$-*b*-BuA$_{300}$-*b*-DiAcAAm$_{10}$, and its two precursors, HDDA$_6$ and HDDA$_6$-*b*-BuA$_{300}$.

Star No. 5

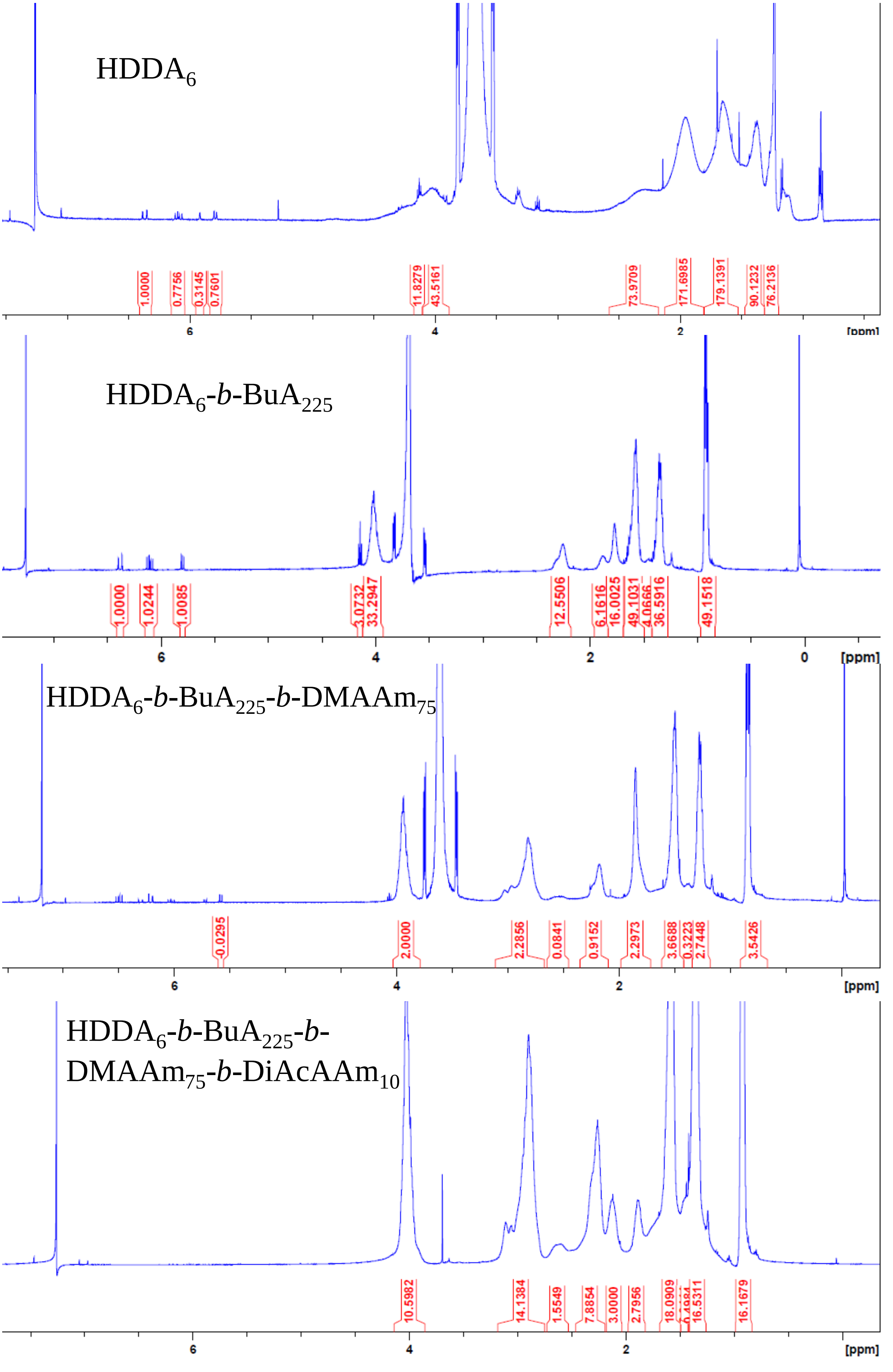

Figure S4. ^{1}H NMR spectra in $CDCl_3$ of the BuA-rich amphiphilic star block “copolymer”, HDDA$_6$-*b*-BuA$_{225}$-*b*-DMAAm$_{75}$-*b*-DiAcAAm$_{10}$, and its three precursors.

Star No. 4

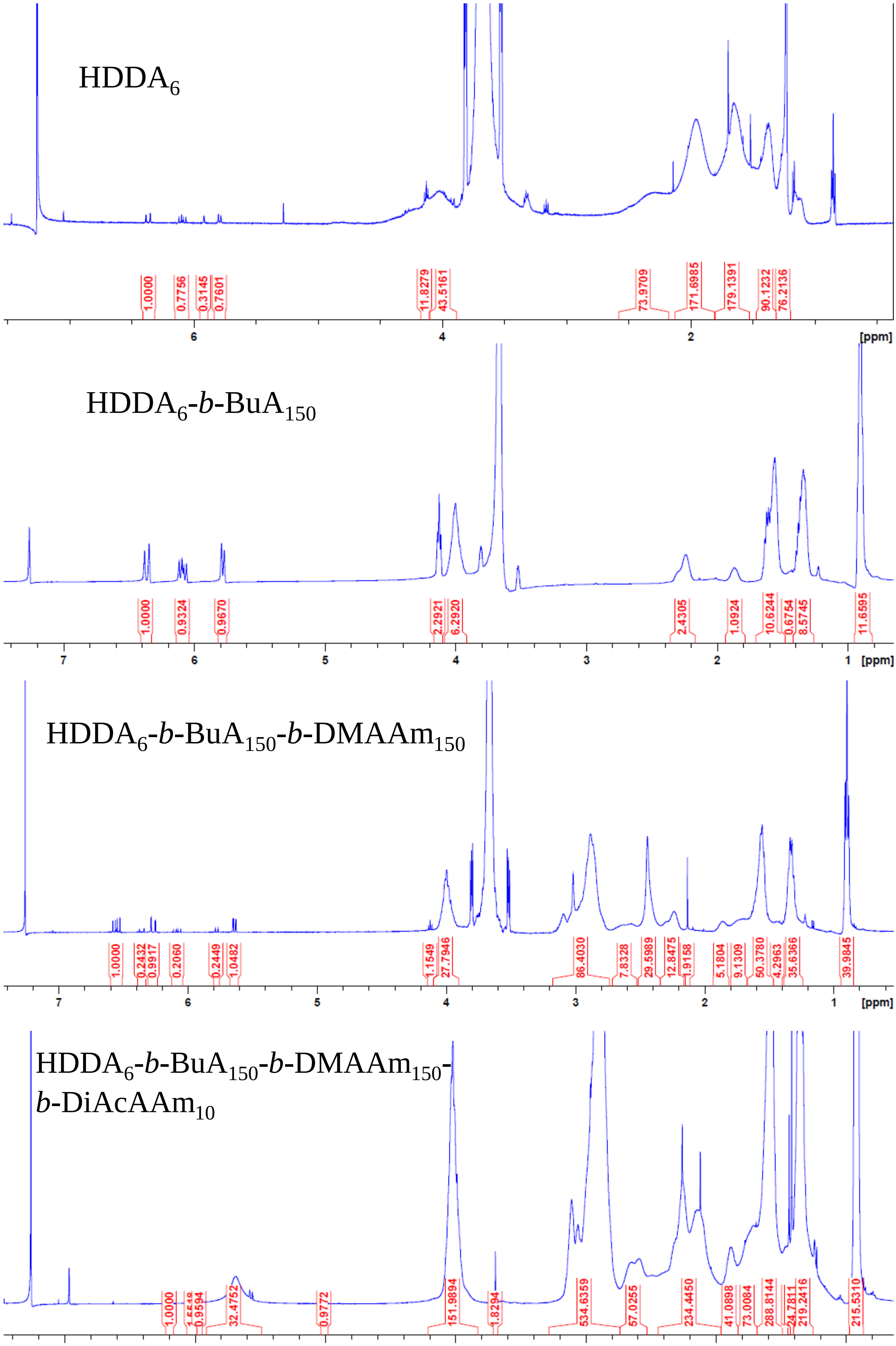

Figure S5. ^{1}H NMR spectra in $CDCl_3$ of the equimolar amphiphilic star block "copolymer", HDDA$_{6}$-*b*-BuA$_{150}$-*b*-DMAAm$_{150}$-*b*-DiAcAAm$_{10}$, and its three precursors.

Star No. 3

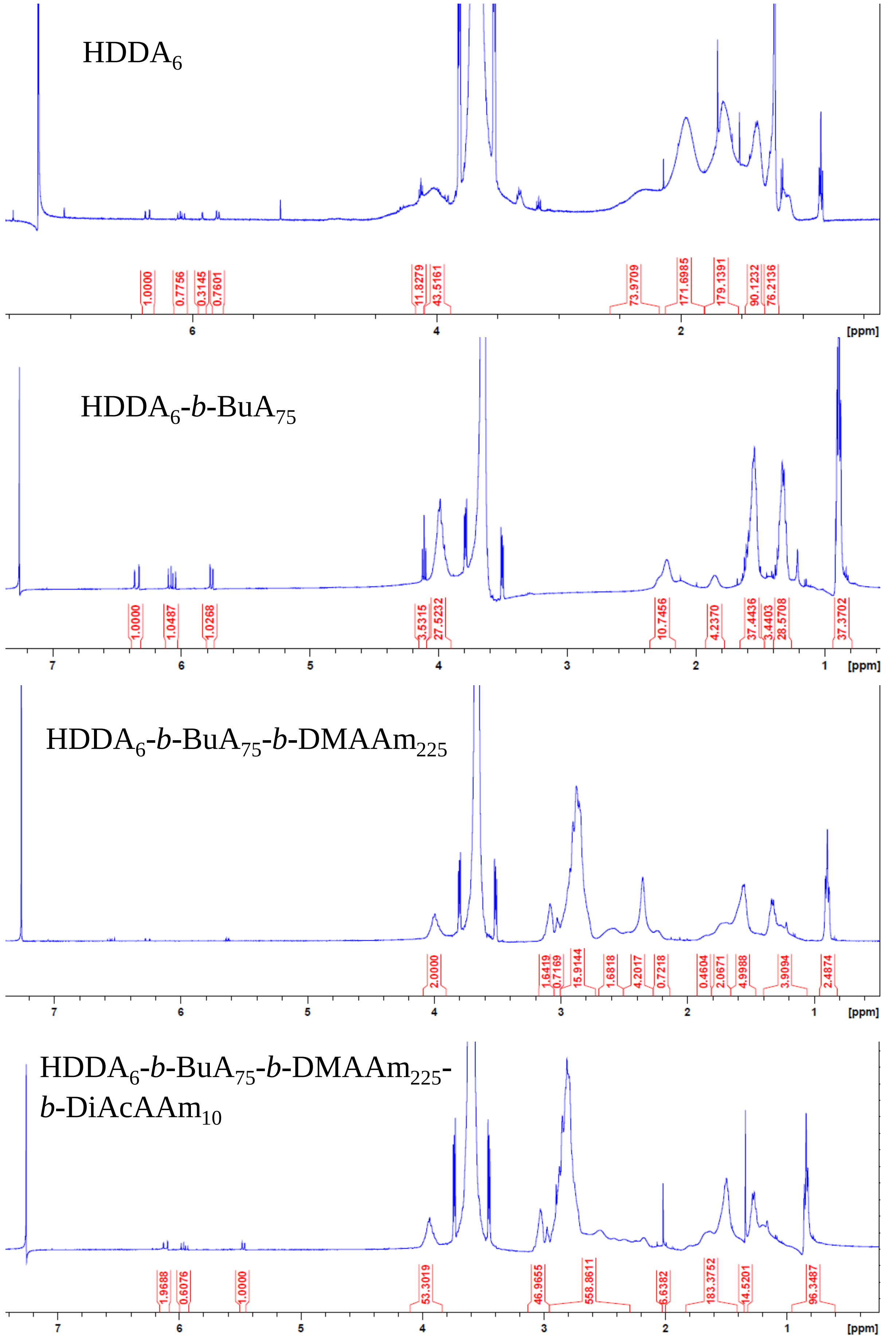

Figure S6. ^{1}H NMR spectra in $CDCl_3$ of the DMAAm-rich amphiphilic star block "copolymer", $HDDA_6$-*b*-BuA_{75}-*b*-$DMAAm_{225}$-*b*-$DiAcAAm_{10}$, and its three precursors.

Star No. 2

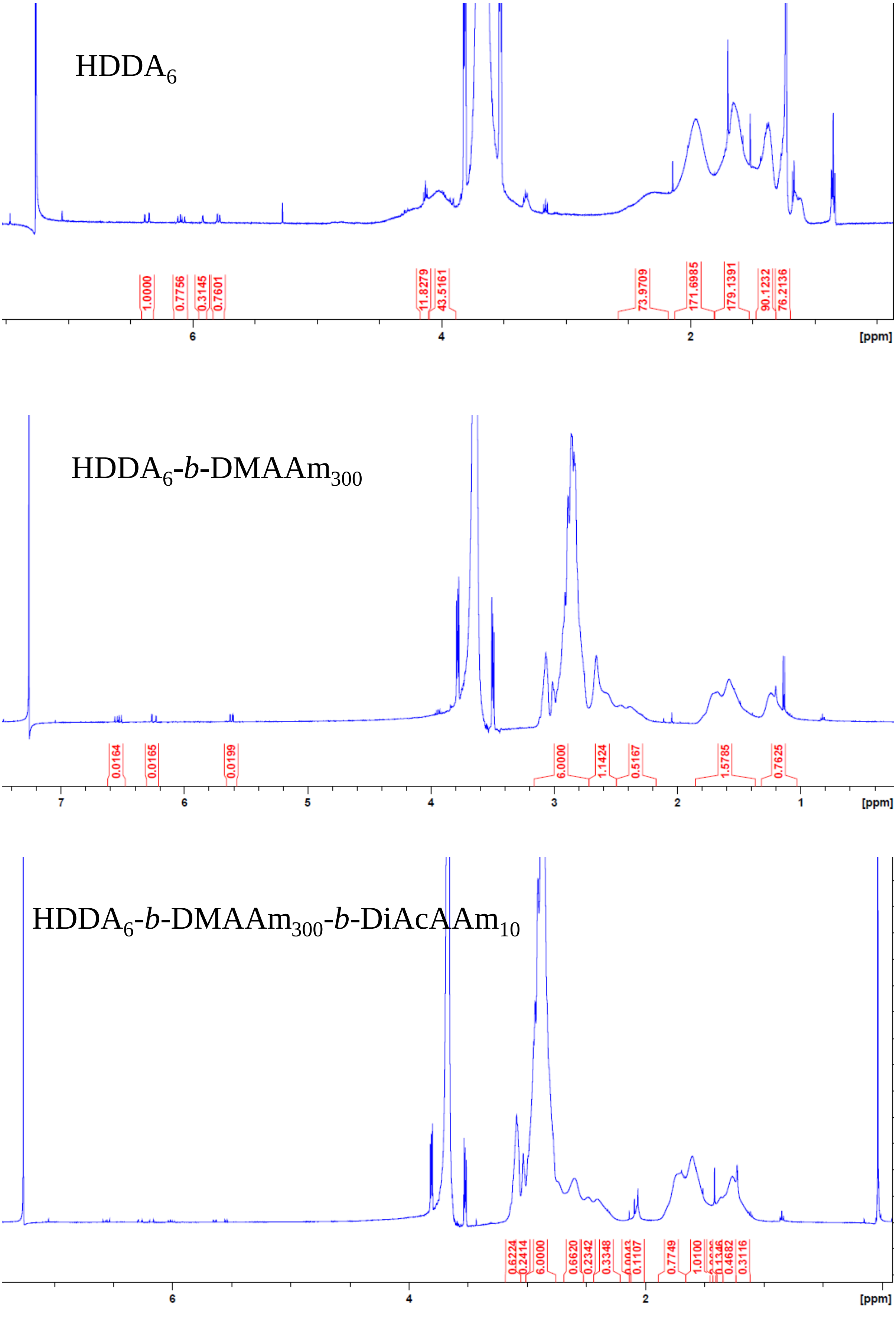

Figure S7. ^{1}H NMR spectra in $CDCl_3$ of the DMAAm hydrophilic star "homopolymer", HDDA$_6$-*b*-DMAAm$_{300}$-*b*-DiAcAAm$_{10}$, and its two precursors, HDDA$_6$ and HDDA$_6$-*b*-DMAAm$_{300}$.
